# Ankle Home Stay Programme:- A review of ankle fracture management and costs at a busy district general hospital

**DOI:** 10.1016/j.amsu.2019.07.020

**Published:** 2019-12-03

**Authors:** Raghbir Khakha, Onur Berber, Amit Patel, Langhit Kurar, Laurence James

**Affiliations:** Department of Trauma and Orthopaedics, University Hospital Lewisham, High Street, Lewisham, London, SE13 6LH, United Kingdom

**Keywords:** Ankle, Fracture, Audit, Cost, Home

## Abstract

**Introduction:**

Patients suffering ankle fractures provide a common economic and time burden to modern healthcare in the UK. They continue to be admitted to await operative intervention and may have to wait days before an operation occurs. Unnecessary bed stay is one are that may be subject to cost savings if the safety of the patient is maintained.

**Patient and methods:**

We prospectively collected data on 23 patients over a four-month period identifying their admission status, length of stay, and time to operative intervention. We were able to cost analyse the patients journey from admission to discharge, postoperative intervention. We then instilled the Ankle Home Stay Programme, identifying patients safe to be discharged who were able to re-attend for their operation. Seventeen patients were enrolled in this and a subsequent cost-analysis was compared to the pre-intervention cohort.

**Results:**

Pre Ankle Home Stay Programme demonstrated cost per patient of admitted patients to be £2690 and £1347 for patients managed in the outpatient setting. Implementation of the pathway maintained this cost saving with those in the outpatient setting costing £1781 per patient and those admitted costing £2855.

**Conclusions:**

Patients can be safely managed in the outpatient setting, with regular clinic review before formal operative intervention as opposed to blanket admission to an acute inpatient bed. This is cost saving in a healthcare system with finite resources focussed on improving use of economic resources. It also maintains patient care with select admission criteria onto the pathway and regular review to ensure standards are maintained.

## Highlights

• Patients can be safely managed in the outpatient setting.
• Expedited home discharge with limited risk to acquiring hospital borne conditions.
• Cost efficiency savings for inpatient stay.

## 1 Introduction

Ankle fractures make up 9% of all fractures [1] and 15% of all ankle injuries [2]. Injuries to the ankle and foot in 2016–2017 accounted for over 17,000 admission in the united kingdom [3].

Costs associated with non-elective hospital stays have steadily increased. In the 2016-2017, the average non-elective inpatient stay was £1590 compared to a day case procedure of £738. This had increased from £1489 and £693 respectively [4]. The rising cost of care is not only an issue for orthopaedic surgery but is an issue across the health service. Opportunities to reduce costs whilst maintaining patient safety must be sought after.

We undertook a prospective review of our current management of ankle fractures before implementing changes for cost-saving and improving patient satisfaction whilst maintaining high standards of patient healthcare.

## 2 Patients and methods

We reviewed our current practice to gauge cost-effectiveness and patient satisfaction over a four-month period from March 2014 to July 2014. We prospectively identified 23 patients who presented to our accident and emergency department with ankle fractures requiring operative intervention (mean age 40.3 years, 9 males, 14 females). Seventeen of these patients were admitted to the orthopaedic ward and five were discharged home. There was no consensus on who was admitted and who was discharged home. One patient who was discharged had subsequent management at another institution.

**Fig. 1:**
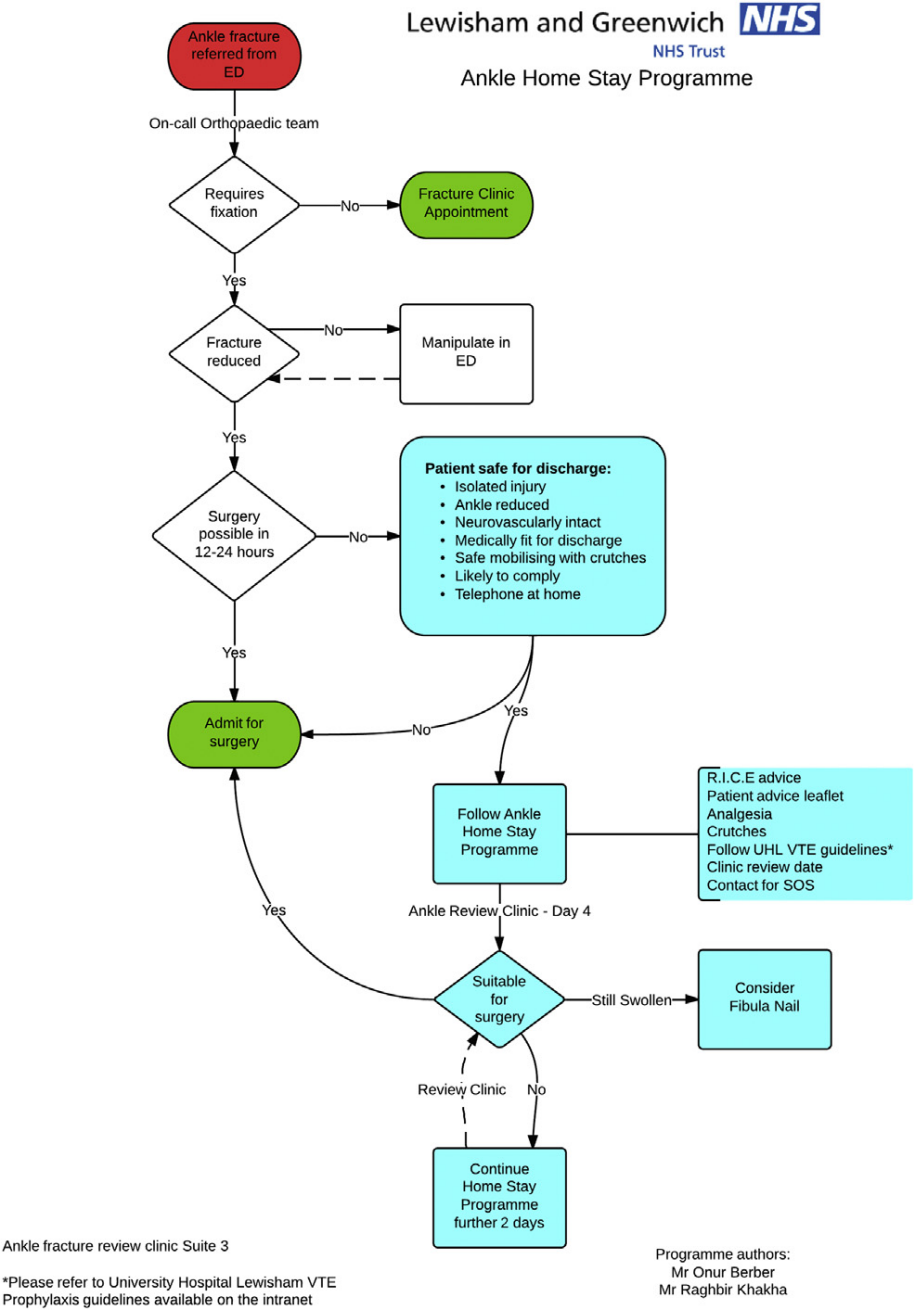
AHSP Flow of patients.

Having established the current practice and their costs, we introduced the Ankle Home Stay Programme (AHSP). This formalised programme consisted of early identification of patients who would be suitable for discharge from accident and emergency to their own home whilst awaiting surgical intervention (Fig. 1).

An orthopaedic clinician reviewed the patient at the time of presentation to accident and emergency, to discuss the AHSP. If time was identified and swelling allowed, the patient was scheduled for early operative intervention the next day and they were admitted. Failing this, patients were discharged home providing they fulfilled our inclusion criteria (Table 1): - these were put in place and adhered to, to ensure the patient was safe for discharge. If they did not fulfil the inclusion criteria, they were admitted to the orthopaedic ward.

**Table 1:** Inclusion criteria.

Isolated Injury
Ankle Reduced
Neurovascularly Intact
Medically Fit for Discharge
Safely Mobilising on Crutches
Likely to Comply
Telephone at Home

Advice was given on resting and elevating the affected limb at home. They were placed in a non-weightbearing below knee plaster of paris cast with crutches and given appropriate analgesia. They were risk analysed for a deep vein thrombosis and prescribed clexane accordingly. They were given a contact number in case of any questions or emergencies, which included the fracture clinic and a trauma coordinator available 24 h a day.

Patients on the programme were booked into a specific plaster technician-led ankle fracture swelling review clinic where the soft tissue envelope was assessed to ascertain suitability for operative intervention. They also received a plain radiograph on the day to monitor for fracture displacement. Each day, an assessment of the trauma schedule for the following day was carried out to identify possible availability for space for operative intervention. If swelling and theatre space allowed, the patient was called into hospital for an operation when a slot was available. This operation was proposed as a day-case intervention.

## 3 Results

Of our preintervention cohort of 22 patients, 17 were admitted (77%) and eight had open reductions and internal fixation. Nine patients had delayed operative intervention at a mean of four days post admission (range: 3–11); with a mean post-operative stay of 4.4 days (range: 1–14 days). For the five patients discharged home, the mean time to operative intervention was seven days (range: 5–12 days), with an average post-operative stay of two days (range: 2-3 days).

Cost-analysis of those who were admitted for their operation and had delayed operative intervention demonstrated a mean cost per patient of £2690. For the five patients discharged home, the mean cost per patient was £1347. These were based on NHS reference costs (Table 2).

**Table 2:** NHS reference costs.

Average cost of a day case	£693
Average cost of a non-elective short or long stay	£1489
Average cost of excess bed-stay	£273
Average cost of an outpatient attendance	£108

After our AHSP was initiated, we reviewed patients managed for an ankle fracture between January 2015 and March 2015. Seventeen patients presented to our accident and emergency department with ankle fractures requiring operative intervention (mean age 45.3 years, 7 males, 10 females). Five (29%) were admitted to an inpatient ward (Table 3). Of these, four were deemed not safe or inappropriate for home management and waited a mean of 9.6 day for their operative intervention (Range 4–18 days) with a post-operative stay of 1.6 days (Range 0–11 days).

**Table 3:** Reasons for admission in re-audit.

Open fracture
Admission post failed manipulation
Social issues
Fall as an inpatient
For early fixation

Twelve patients went on to follow the AHSP pathway. Two were readmitted from this pathway to be inpatients:- one for slipped position in a backslab and another for an unacceptable reduction in accident and emergency the previous day. The admitted patients on this cohort demonstrated a cost of 2855 per day whilst those whom remained on the home wait pathway cost £1781 per day.

## 4 Discussion

Our review of ankle fracture management shows that managing patients at home pre-operatively is more cost-effective than blanket admission to an acute orthopaedic bed. In modern health-care, cost saving is the responsibility of all health care individuals; it is vital to find areas where one can safely implement programmes similar to ours while maintaining patient safety and high satisfaction. The benefits of our programme allow patients to await surgery in the comfort of their own home while having the safety net of regular clinic follow-up.

Apart from this, there are knock on positive effects. A reduction of admissions from accident and emergency unnecessarily to inpatient wards creates free beds for other patients. This reduces wait times in accident and emergency and allows a better flow of patients through the hospital system. There is also a reduction in inpatient waiting times. The programme also encourages regular interaction between the orthopaedic and emergency department. Subsequently, a regular teaching programme was formed to educate the Emergency Department three times a year as their trainees rotated through.

The added cost in our programme was the visit to clinic to assess for swelling. Patients on the programme did not replace another patient on a fracture clinic schedule. They were scheduled as part of a plaster room led clinic, where a senior registrar or consultant was present to have a look at the swelling once plaster technicians had removed the plaster. This did not result in a significant burden to the clinic schedule and was well received by other member of the fracture clinic team.

There was no increase in adverse events as a result of implementing this programme. We did not detect any increase in thromboembolic phenomenon, though we continue to monitor this due to the short time frame.

Formal management of patients at home has been advocated by a study from Dorset hospital [5]. Here, their home management programme incorporates physiotherapists calling patients to ascertain how they were doing. If any issues arose they were invited to hospital for clinical review. Otherwise the patient was next seen at the time of their operation. We felt it was important for regular clinical review as the assessment of the soft tissue envelope is key and is based on a clinical subjective examination. Discussing issues over the phone in our opinion would have been suboptimal. We also picked up one patient whose fracture had slipped position and required admission. This again would not have been possible with purely telephone contact. Subtly non-congruent ankle joints or grossly dislocated ones can compromise the time taken for swelling to get better, exacerbate pain and on rare occasions may need management with an external fixation system. We accept that regular clinical review could also incur transport costs, which are all an addition to the overall cost of the patient's care episode. We did not specifically factor looking into this as part of our study.

The modern approach is for early fixation (before 24 h) to negate the wound complications associated with fixation of a swollen ankle. As a result, the added benefits include avoiding the prolonged delay while waiting for swelling to be appropriate. Evidence suggests that this early intervention leads to a reduced inpatient postoperative stay [6]. Unfortunately, a combination of high trauma workload and delayed presentation means early operative intervention is rarely possible. Subsequently, patients remain as inpatients, awaiting a reduction in swelling, before having their operation. In some instances, this can take up to 2 weeks.

Murray et al. [7] recommended that managing the patient at home whilst the swelling settles is a viable option in this patient cohort. Their study demonstrated that longer admissions were costlier based on those patients having external fixation as a primary procedure, multiple procedures or patients having severe health problems. Longer admissions will incur larger costs. Length of inpatient stay is multifactorial:- swelling, trauma workload, comorbidities, are only some of the factors that can have an impact. Kheir et al. [8] compared three subcohorts of ankle fracture patients:- early fixation vs elevation and delayed fixation vs application of external fixator and delayed fixation. Predictably the early fixation group had the lowest inpatient stay and incurred the least financial costs.

A reduction in unnecessary admissions also lessens the risk of patients contracting hospital acquired illness as a result of their admission which affects in particular, elderly and frail people who may have a fragility related ankle fracture and many co-morbidities. High comorbidities translate to a higher American Society of Anaesthesia (ASA) score, which studies have shown to be an indicator to length of stay [9]. Change of environment into inpatient hospital wards can have psychosocial consequences. Frail, elderly patients can experience compound delirium on pre-existing dementia. Younger patients also have a preference for management in their own home, thus lessening the impact of becoming a ‘patient’.

## 5 Conclusion

Our study demonstrates that initially managing ankle fracture patients at home, is more cost-effective than admitting all patients to the hospital to await their operations. It reduces bed pressures to hospitals. It also reduces the risk of inpatient stay associated mortality. Although some hospitals may already manage their patients in a similar way, formalisation of this pathway is key for maintaining standards and audit. The addition of an ankle review clinic ensures regular patient review for assessment of swelling and late displacement.

## Ethical approval

No ethical approval required.

## Sources of funding

No sources of funding.

## Author contribution

Raghbir Khakha - Consultant Trauma and Orthopaedic Surgery.

Onur Berber – Consultant Trauma and Orthopaedic Surgery.

Amit Patel - Specialist Registrar Trauma and Orthopaedic Surgery.

Langhit Kurar - Specialist Registrar Trauma and Orthopaedic Surgery.

Laurence James - Consultant Trauma and Orthopaedic Surgery.

All authors have made substantial contributions to all of the following:

(1) the conception and design of the study, or acquisition of data, or analysis and interpretation of data
(2) drafting the article or revising it critically for important intellectual content
(3) final approval of the version to be submitted

## Registration of research studies

ISRCTN94661773.

## Guarantor

Amit Patel - Specialist Registrar Trauma and Orthopaedic Surgery.

Langhit Kurar - Specialist Registrar Trauma and Orthopaedic Surgery.

## Consent

Not applicable.

## Declaration of competing interest

No conflicts of interest.
